# Autoantibodies against CYP-2C19: A Novel Serum Marker in Pediatric De Novo Autoimmune Hepatitis?

**DOI:** 10.1155/2017/3563278

**Published:** 2017-11-27

**Authors:** Maria Grazia Clemente, Roberto Antonucci, Claudia Mandato, Lucia Cicotto, Antonella Meloni, Bruno Gridelli, Stefano De Virgiliis, Michael P. Manns, Pietro Vajro

**Affiliations:** ^1^Department of Biomedical Sciences and Biotechnologies, 2nd Pediatric Clinic, University of Cagliari, Cagliari, Italy; ^2^Pediatric Clinic, Department of Surgical, Microsurgical and Medical Sciences, University of Sassari, Sassari, Italy; ^3^Pediatrics, Department of Medicine, Surgery, and Dentistry “Schola Medica Salernitana”, University of Salerno, Baronissi, Salerno, Italy; ^4^Pediatrics Unit, AORN Santobono-Pausilipon, Naples, Italy; ^5^Mediterranean Institute for Transplantation & Advanced Specialized Therapies (ISMETT), Palermo, Italy; ^6^Department of Gastroenterology, Hepatology and Endocrinology, Medical School Hannover, Hannover, Germany

## Abstract

Diagnosis of de novo autoimmune hepatitis (AIH) after orthotopic liver transplantation (OLT) is challenging especially in the absence of hyper-*γ*-globulinemia. Circulating autoantibodies are not sensitive nor specific in de novo AIH but when positive increase the diagnostic probability. We report the discovery of novel liver microsomal (LM) autoantibodies against CYP-2C19 in a 9-year-old boy with “de novo” AIH developed 7 years after OLT. Graft dysfunction presented with hypertransaminasemia (up to 400 IU/L), while serum *γ*-globulins remained within the normal range for age. Liver histology and response to high dose prednisone (2 mg/kg/day) with the addition of azathioprine therapy further supported the diagnosis of de novo AIH. Autoantibodies investigation by indirect immunofluorescence (IF) on rodent tissues showed a novel staining pattern involving the pericentral liver zone and sparing the renal tissue. Human but not rat liver proteins immunoblotting allowed us to characterize the novel LM antibodies and to identify CYP-2C19 as human antigen. The finding offers insights into the controversial discussion about autoimmunity versus alloreactivity with regard to the pathogenesis of de novo AIH. Correct information on human versus rat tissue antigens tested by methods other than IF for antibodies detection may have significant implications for the correct diagnosis and management of patients followed up after OLT.

## 1. Introduction

De novo autoimmune hepatitis (AIH) after orthotopic liver transplantation (OLT) should be suspected in any unexplained graft dysfunction, in both children and adults transplanted for an indication different from autoimmune liver diseases [1, 2]. It is still not clear whether the pathogenesis is autoimmune directed against the “self” or alloimmune against the “non-self” after OLT. Passive transfer of autoantibodies from the organ donor, induction by viral infection, drug therapy, and ischemic injury of rejected transplanted organ are the main hypothesized mechanisms [1, 2].

Differential diagnosis with the far more common graft rejection and viral hepatitis is challenging in spite of the diagnostic criteria for de novo AIH proposed in 2006 by the Banff working group [3].

Both typical and atypical circulating autoantibodies have been found to be associated with de novo AIH. Among the typical ones [4, 5], anti-nuclear antibodies (ANA) have been the most frequently detected (42.8%–100%), followed by anti-smooth muscle antibodies (SMA) (25%−50%), while anti-liver/kidney microsomal antibodies (LKM) have been detected more rarely. Atypical LKM antibodies are peculiar for de novo AIH. They have been renamed anti-liver/kidney cytosol (LKC) because they do not react with the liver microsomes but with the cytosol and are specifically directed against the cytosolic enzyme glutathione-S-transferase theta 1 (GTT1) [6, 7]. Autoantibodies against cytokeratins 8 and 18 (CK8-18) have been also detected in subjects with de novo AIH [1, 8].

Here, we report for the first time in a child with de novo AIH cytochrome P450 2C19 (CYP-2C19) as a hepatocellular autoantigen of new liver microsomal (LM) autoantibodies. LM against CYP-2C19 are different from the previously described LM autoantibodies directed against CYP-1A2, considered hallmark of AIH in children affected by Type 1 Autoimmune Polyglandular Syndrome (APS 1) [9–16].

## 2. Patient and Methods

### 2.1. Patient and Controls

The case is a 9-year-old boy who developed evidence for de novo AIH, seven years after OLT for biliary atresia. Controls included 50 healthy subjects and 66 nontransplanted patients affected by idiopathic autoimmune liver diseases: 11 with AH-1 (ANA and/or SMA positives), 11 with AH-2 (LKM-1 and/or LC-1 positives), 2 with AH-3 (SLA positives), 26 with Primary Biliary Cholangitis (PBC, AMA positives), and 16 with APS-1 (one LM positive, five LKM positives).

### 2.2. Indirect Immunofluorescence (IF)

Rat kidney, liver, and stomach tissue sections were used for ANA, SMA, LKM, and AMA detection (SCIMEDX, Denville, New Jersey, USA), and human epithelial type 2 (HEp-2) cells were used for ANA, AMA, and anti-actin antibodies detection (MarDx diagnostics, Carlsbad, California, USA). Briefly, slides were incubated in moist chamber for 30 minutes with serum serial dilutions (from 1 : 40 to 1 : 1280) at room temperature (RT). Then they were washed twice for 5 minutes in phosphate buffer saline (PBS) solution, incubated at RT for 30 minutes with FITC-conjugated goat anti-human sera (IgA + IgG + IgM, Medic, Castello di Pavone, TO, Italy), diluted 1 : 100 in PBS, washed twice as before, and read with a fluorescence microscope (Olympus BX51).

### 2.3. Immunoblotting (IB) with Rat and Human Liver Proteins

IB experiments were carried out using both rat and human liver tissues. Human liver was obtained during liver transplantation from a reduced size donor liver whose material in excess would be anyway discarded. Microsomal, mitochondrial, and soluble protein fractions from rat and human liver were prepared by differential centrifugations and ultracentrifugations as described previously [11]. Rat and human liver subcellular protein fractions (microsomal, mitochondrial, and soluble) were separated in different experiments. Polyacrylamide gel electrophoresis (PAGE) was carried out under denaturing conditions for rat proteins by sodium dodecyl sulfate-PAGE (SDS-PAGE, 12%) while human proteins were separated under not denaturing conditions PAGE (12%) in absence of SDS. After electrophoresis, both rat and human proteins were transferred from the gels onto nitrocellulose filters as described previously [11, 12]. Filters were blocked for 1 hour in a PBS 0.1% Tween-20, 3% nonfat dry milk solution, incubated for 1 hour at RT with patient sera at 1 : 100 dilution, and successively incubated for 1 hour at RT with 1 : 1000 dilution of alkaline phosphatase conjugated anti-human IgG + IgM + IgA antiserum (ICN Biomedicals, Aurora, Ohio, USA). The NBT-BCIP detection system (Bio-Rad, Hercules, CA, USA) was used to develop the filters.

### 2.4. Immunoblotting (IB) with Six Human Recombinant CYPs and UGT1

Baculovirus insect cell-expressed human hepatic recombinant cytochrome P450s and UGT1 were purchased from Gentest Corp. (Woburn, MA, USA) [12]. The IB assay used was SDS-PAGE (12%), following the steps described in Section 2.3.

## 3. Results

### 3.1. Clinical Report

A 9-year-old boy developed de novo AIH 7 years after liver transplantation for biliary atresia. He was under treatment with cyclosporine (CyA) (trough blood levels of 151 *μ*g/L). The immune-mediated graft dysfunction occurred during prednisone (PDN) tapering/weaning dose (from 5 mg to 0 mg/day) and presented with a progressive increase of serum ALT (from <40 IU/L up to 400 IU/L) and IgG (from 600 mg/dl up to 1200 mg/dl). Liver biopsy revealed a histological picture of portal tracts enlarged by a dense lymphoplasmacytic infiltrate and fibrosis, moderate interface hepatitis with intralobular inflammation, and necrosis in absence of relevant evidence for rejection (data not shown). Different possible causes of liver dysfunction other than graft rejection and AIH were ruled out by appropriate tests, including serology and molecular testing for hepatitis B virus, hepatitis C virus, Cytomegalovirus, Epstein Barr virus, and parvovirus B19; moreover, sonography and color Doppler sonography were performed to rule out biliary and vascular abnormalities. Globally, the patient diagnostic score was within the “probable” range of AIH. Both ALT and IgG serum levels promptly responded (<40 U/L and 800 mg/dl, respectively) to the increase in the PDN dosage up to 2 mg/kg/day and to additional treatment with azathioprine (1 mg/kg/day), further supporting the diagnosis of de novo AIH. During a 30-month-follow-up period, the patient relapsed after two attempts of PDN tapering dose from 10 to 5 mg/day.

### 3.2. Indirect Immunofluorescence (IF)

**Figure 1:**
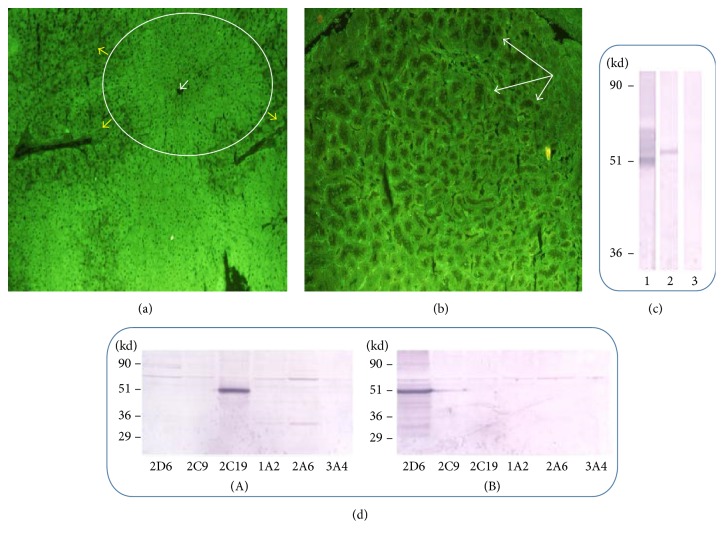
(a-b) Indirect immunofluorescence (IF) results using the patient serum diluted at 1 : 640 in phosphate buffered saline and overlaid on rat liver (a) and kidney (b) sections (photomicrograph; lens 10x). For immune staining, a goat anti-human Ig FITC-conjugate antiserum was used at 1 : 100 dilution. The new LM antibody staining pattern involved mainly hepatocytes of the pericentral zone ((a), white arrow points to the central vein) and extended to the mid-lobule zone of the liver ((a), white circle, yellow arrows indicate the surrounding negative zone) while sparing the renal proximal tubular cells ((b), white arrows). (c) Immunoblotting results using human liver microsomal proteins separated in PAGE. Lane 1, LKM positive control serum from a patient affected by type 2 idiopathic AIH, main protein band recognized at ~50 kD; lane 2, LM positive serum from the reported case affected by de novo AIH, main protein band recognized at >51 kD; lane 3, atypical LKM positive control serum from a post-OLT patient, no protein band recognized. Markers on the left indicate the molecular weight standards in kilodaltons (kD). (d) Immunoblotting results using six recombinant cytochromes P450. Lanes from the left to the right: CYP-2D6, CYP-2C9, CYP-2C19, CYP-1A2, CYP-2A6, and CYP-3A4. (A) Results observed using the LM positive serum from the reported case affected by de novo AIH, autoantigen recognized: CYP-2C19. (B) Results observed using a LKM positive control serum from a patient affected by type 2 idiopathic AIH, autoantigen recognized: CYP-2D6. Markers on the left indicate the molecular weight standards in kilodaltons (kD).

The patient serum was negative for typical ANA, SMA, and LKM autoantibodies associated with AIH. IF analysis showed a novel pattern, involving prevalently the pericentral zone and extending to the mid-lobule zone of the liver (Figure 1(a)), while sparing the staining of the renal proximal tubular cells (Figure 1(b)). This new IF pattern appeared distinct from that of atypical LKM or LKC pattern which is characterized by hepatic and renal proximal tubular cell fluorescence immunostaining [1], detected also by our group in 3 out of 45 post-OLT cohort of children [19].

### 3.3. Immunoblotting (IB) with Rat and Human Liver Proteins

While no reactivity was observed in experiments with rat liver proteins (data not shown), a positive reaction with a protein band was observed above the 51 kD molecular weight standard in IB experiments performed with the microsomal fraction of human liver (Figure 1(c)). No reactivity appeared toward the other two liver protein fractions, the soluble and the mitochondrial, of human liver (data not shown). The peculiar aspects observed at IF and IB assays allowed us to classify the novel antibodies as liver microsomal (LM).

### 3.4. Immunoblotting (IB) with Six Human Recombinant CYPs and UGT1

In order to identify the main target antigen of the novel LM antibodies observed in IF, we performed IB analysis using several commercially available human recombinant CYPs and UGTs (BD Gentest, http://www.bdbiosciences.com) as previously described [12]. The serum that was positive in IF for LM antibodies showed specific reactivity with only one of the human recombinant CYPs tested (Figure 1(d)), namely, the CYP-2C19, a protein of 55,9 kD molecular weight (NCBI number: NP_000760.1). CYP-2C19 is one of the human liver microsomal enzymes belonging to the 2C family of the cytochrome P450 superfamily. The same serum did not react with any other CYPs and UGTs (Figure 1(d)). During the 30 months of follow-up for de novo AIH, LM antibody titer significantly changed, decreasing to negative under the max dose of immunosuppression, while coming back at any flare of relapse.

Atypical LKM positive sera of our previously described post-OLT patients [19] did not react against any of the CYPs or UGTs, including CYP-2C19 (data not shown).

CYP-2C19 was then tested as a potential antigen in 24 control sera from patients with idiopathic AIH, 26 patients with PBC, 16 patients with APS-1, and 50 healthy controls. None of these sera showed a positive reaction with the CYP-2C19 (data not shown).

## 4. Discussion

The precise mechanism underlying de novo AIH is still obscure, and controversy exists about autoimmunity versus alloreactivity for explaining this immune-mediated graft dysfunction that can arise after an allogenic organ transplantation [20]. On the other hand, a graft rejection after OLT may mimic de novo AIH [20].

Circulating autoantibodies are a common finding after OLT especially in children [1]. However, in some cases, serum IgG levels or autoantibodies can be normal or absent, respectively, making the understanding and the diagnosis of de novo hepatitis quite challenging [1, 18, 21].

Our case had most of the features of de novo AIH, including increasing serum *γ*-globulins, even if the values remained always within the normal range of reference for his age. He was positive for circulating autoantibodies. At IF screening, however, their fluorescence immunostaining pattern appeared distinct from that of atypical LKM or LKC, whose target is GSTT1, and from antibodies against CK 8-18 previously described in other series of de novo AIH [6–8]. The IB assay performed with human liver allowed us to characterize the novel autoantibodies as LM because it is directed toward the microsomal protein fraction and toward a band identified as CYP-2C19.

To the best of our knowledge, LM in children has hitherto been described only in the context of APS1-related AIH [9–16]. We therefore carried out a review of literature data on LM and LKC autoantibodies laboratory characterization performed with at least one adjunctive assay besides traditional IF screening. The results are shown in Table 1. During the past 25 years, nine articles have been published. The studies on the characterization of LM autoantibodies reacting against the hepatic CYP-1A2 regarded essentially adults suffering from hydralazine-induced AIH [17] or children and adults affected by AIH as part of APS-1 [9–16]. These studies used the same assay represented by IB of human proteins to identify the target antigen, that is, human CYP-1A2. The three studies published on the characterization of LKC autoantibodies were carried out in adults suffering from de novo AIH. The first study successfully identified GSTT1 as target antigen of LKC by testing positive sera with human liver cDNA expression library [6], the second used immunodiffusion assay exclusively with rat liver cytosol proteins [18], and the third one used two-dimensional IB assay performed with rat proteins to characterize both LKC and classical LKM-1 autoantibodies [7].

Similar to what has happened in the CYP-1A2 identification, in the present study, CYP-2C19 could be identified as LM target antigen when IB was performed with human liver microsomes, although the IF was performed on rodent tissue. This apparent discrepancy of LM antibodies has been explained by differences existing between the rat and human CYPs' structure [11, 17]. Rat CYPs would share only a few conformational epitopes with the human enzyme, which are preserved in IF but are lost during sample preparation in IB [11, 17]. It implies that, in the case of rat tissue preparation only, our patient could have been un/misdiagnosed, because besides a positive IF we would have found a misleading negative IB.

As the target antigen is an enzyme of the cytochrome P450 family, the novel LM antibodies detected in our case are unlikely donor specific antibodies (DSA). DSA react either to donor lymphocytes or to a panel of HLA antigen targets; their production derives from a mismatch in the liver expressed proteins [22]. DSA can lead to an alloimmune hepatitis and have been reported to cause antibody mediated acute rejection developing de novo after transplantation [1, 22]. Some studies reported high levels of DSA in up to 22% of liver transplant recipients without increased incidence of acute rejection [22].

A post-OLT graft dysfunction mimicking de novo AIH has also been described when GSTT1 is an alloantigen becoming a* neo*-self-antigen due to a mismatch between the liver donor and recipient [23]. Rising against the “non-self” liver, this graft dysfunction can be defined as an alloreactive immune response rather than an autoimmune one. Subjects who genetically do not express GSTT1 may develop an immune reaction against GSTT1 after OLT, detected serologically with the appearance of atypical LKM or LKC antibodies [23]. The “non-self” alloreactive type of immune response has been ruled out for anti-LKM-1 antibody, whose serum positivity has been reported in genetically CYP-2D6 extensive metabolizer individuals affected by AIH-2 [5, 24], in whom a sufficiently high CYP-2D6 expression level is thought to be necessary to trigger autoimmunity [5].

Since about 3% of Caucasians lack CYP-2C19 entirely [25], it could be speculated that, rather than a true AIH, our patient developed an immune reaction against a “non-self” protein, that is, CYP-2C19, expressed in the graft. Unfortunately, we could neither test whether our patient and the graft were genetically different with regard to CYP-2C19 to verify the existence of a CYP-2C19 mismatch between donor and recipient, nor test whether the CYP-2C19 was expressed and functionally active in the native liver of our patient similarly to CYP-2D6 in AIH-2 patients [24].

Several human liver cytochrome P450s have been reported as autoantigens of the liver/kidney or liver microsomal (LKM or LM) autoantibodies associated with chronic hepatitis of different etiologies, with very little overlap [4]. The IF pattern of each microsomal autoantibody will depend on the specific target antigen, whose expression can be variable from the pericentral to the periportal zone of the hepatic lobule [4, 5]. It is important to note the report of liver microsomal autoantibodies detected at IF for which target antigens have been not yet identified [5]. Therefore, the identification of CYP-2C19 as a human hepatic autoantigen adds one more enzyme to the existing list.

In spite of the continuous progress in discovering new autoantibodies and new autoantigens, etiology and pathogenesis are clear only for the drug-induced forms of AIH. By binding covalently to its specific metabolizing enzyme, the drug creates a new molecule that acts as a neoantigen and triggers the autoimmune response in genetically predisposed individuals [26]. CYP-2C19 is one of the most important microsomal enzymes involved in hepatic drug biotransformation reactions [25]. The commonly used omeprazole, several benzodiazepines, and many of the tricyclic antidepressants are metabolized by CYP-2C19 [25]. Rifampin induces CYP-2C19 activity, while the antidepressants fluvoxamine, fluoxetine, and the antithrombotic Ticlopidine inhibit it [25]. In our case, however, the post-OLT history was negative for the assumption of any drug known to be metabolized by CYP-2C19. After liver transplantation, our patient underwent treatment with cyclosporine, a drug metabolized by CYP-3A4, which was tested negative in our IB experiments, making it unlikely that the novel LM antibodies were cyclosporine-induced.

## 5. Conclusions

The present study is the first report on novel LM autoantibodies directed against CYP-2C19 in a child with de novo AIH. Correct information on human versus rat tissue antigens tested by methods other than IF for antibodies characterization may have significant implications for the correct diagnosis and management of patients followed up after OLT.

We were, however, not able to clarify whether the novel LM autoantibodies here described are marker of autoimmune reactions against the “self” or they derived from alloimmune reactions against the “non-self” after OLT. This study represents a pathfinder on this topic and warrants exploration in more extensive future studies.

## Figures and Tables

**Table 1:** Studies investigating LM and LKC autoantibodies with more than one immunological assay in patients affected by AIH of different etiologies. Studies are ordered by year of publication.

Reference	Year	Diagnosis	Ab	Assay: IF on liver/kidney	Assay other than IF	Target Ag (kD)	Age of patients
Bourdi et al. [17]	1990	AIH-drug (dihydralazine induced)	LM	Liver [**+**] Kidney [−]	IB, rat [−] IB, human [**+**]	CYP-1A2 (~52 kD)	Adult

Sacher et al. [9] Manns et al. [10]	1990	AIH-APS-1	LM	Liver [**+**] Kidney [−]	IB, human [**+**]	CYP-1A2 (~52 kD)	Pediatric

Clemente et al. [11, 12]	1997	AIH-APS-1	LM	Liver [**+**] Kidney [−]	IB, rat [−] IB, human [**+**]	CYP-1A2 (~52 kD)	Pediatric

Gebre-Medhin et al. [13]	1997	AIH-APS-1	LM	Liver [**+**] Kidney [−]	IB, human [**+**]	CYP-1A2 (~52 kD)	Pediatric

Obermayer-Straub [14]	2001	AIH-APS-1	LM	Liver [**+**] Kidney [−]	IB human CYPs	CYP 1A2	Adult and pediatric

Aguilera et al. [6]	2001	Post-OLT AIH	LKC	Liver [**+**] kidney [**+**]	Human liver cDNA expression library [**+**]	GSTT1 (~29 kD)	Adult

Salcedo et al. [18]	2002	Post-OLT AIH	LKC	Liver [**+**] kidney [**+**]	ID, rat [+] Human not tested	Cytosol Ag not studied	Adult

Huguet et al. [7]	2007	Post-OLT AIH	LKC	Liver [**+**] Kidney [**+**]	2D-IB Rat [+] Human not tested	Cytosol Ag = GSTT1 (~25 kD)	Adult

Meloni et al. [16]	2012	AIH-APS-1	LM	Liver [+] Kidney [−]	IB human CYPs	CYP1A2	Pediatric

Clemente et al.	Present study	Post-OLT AIH	LM	Liver [+] Kidney [−]	IB, rat [−] IB, human [+]	CYP-2C19 (55,9 kD)	Pediatric

Ag, antigen; AIH, autoimmune hepatitis; AIH-drug, drug-induced AIH; AIH-APS-1, Type 1 Polyglandular Syndrome associated AIH; CYP, cytochrome P450; GSTT1, glutathione-S-transferase T1; IB, immunoblotting; 2D-IB, two-dimensional immunoblotting; ID, immunodiffusion; IF, immunofluorescence; kD, kilodaltons of Ag molecular weight; LKC, liver kidney cytosol antibody; LM, liver microsomal antibody; LKM, liver and kidney microsomal antibody; post-OLT AIH, postorthotopic liver transplantation “de novo” AIH; Pts, patients.
